# Molecular Identification of Zoonotic Parasites of the Genus *Anisakis* (Nematoda: Anisakidae) from Fish of the Southeastern Pacific Ocean (Off Peru Coast)

**DOI:** 10.3390/pathogens9110910

**Published:** 2020-11-03

**Authors:** Renato Aco Alburqueque, Marialetizia Palomba, Mario Santoro, Simonetta Mattiucci

**Affiliations:** 1 Department of Public Health and Infectious Diseases, Section of Parasitology, Sapienza University of Rome, P.le Aldo Moro, 5, 00185 Rome, Italy; renato.aco.alburqueque@gmail.com (R.A.A.); marialetizia.palomba@uniroma1.it (M.P.); 2 Faculty of Veterinary Medicine and Zootechnics, Universidad Peruana Cayetano Heredia, Av. Honorio Delgado 430, Lima 31, Peru; 3 Laboratory Affiliated to Istituto Pasteur Italia-Fondazione Cenci-Bolognetti, Viale Regina Elena, 291, 00161 Roma, Italy; 4 Department of Integrative Marine Ecology, Stazione Zoologica Anton Dohrn, Villa Comunale, 80121 Naples, Italy; mario.santoro@szn.it

**Keywords:** *Anisakis pegreffii*, *Anisakis* sp. 2, Peru coast, parasitic burden, seafood safety

## Abstract

The study aims to perform, for the first time, the molecular identification of anisakid larvae in commercial fish from the Southeastern Pacific Ocean off the Peru coast, and to provide data on their infection level by fishing ground, fish host, and site of infection. Fish specimens (N = 348) from the northern and the central coast of Peru were examined for parasites. The fish fillets were examined by the UV-press method. *Anisakis* spp. larvae (N = 305) were identified by mtDNA *cox*2 sequences analysis and by the ARMS-PCR of the locus *nas*10 nDNA. Two hundred and eighty-eight *Anisakis* Type I larvae corresponded to *Anisakis pegreffii,* whereas 17 *Anisakis* Type II larvae clustered in a phylogenetic lineage distinct from *Anisakis physeteris* deposited in GenBank, and corresponding to a phylogenetic lineage indicated as *Anisakis* sp. 2, previously detected in fish from both Pacific and Atlantic waters. *Anisakis pegreffii* was found to infect both the flesh and viscera, while *Anisakis* sp. 2 occurred only in the viscera. The average parasitic burden with *A. pegreffii* in the examined fish species from the two fishing grounds was significantly higher than that observed with *Anisakis* sp. 2. The results obtained contribute to improve the knowledge on the distribution and occurrence of *Anisakis* species in Southeastern Pacific waters and their implications in seafood safety for the local human populations.

## 1. Introduction

Industrial fishery represents an important economic sector in Peru. With an estimated production of around 7.4 million tons per year, it is a relevant food resource, and thus offers employment for the coastal populations of the Southeastern Pacific Ocean [1]. In Peru, the industrial fishery has been traditionally based on marine pelagic species, mainly constituted by anchovy (*Engraulis ringens*), as well as in other fish species such as jack mackerel (*Trachurus murphyi*), chub mackerel (*Scomber japonicus*), and Pacific bonito (*Sarda chilensis*) [1]. However, in recent years, there has been an effort to diversify the fishery into other resources including the jumbo squid (*Dosidicus gigas*), dolphinfish (dorado/perico) (*Coryphaena hippurus*), palm ruff (*Seriolella violacea*), and Pacific pomfret (*Brama japonica*) [1]. These last fish species are well distributed along the Peruvian coast, and they also represent the most highly consumed seafood by local populations [2]. In these terms, the higher demand for seafood products, more often consumed even raw, raise the concern about seafood safety and quality [3]. Among the parasites affecting seafood products, the presence of *Anisakis* spp. larvae have an impact for the food safety and human health [4]. The zoonotic implications associated with these parasites are a major concern, and their presence in seafood products, even when worms are dead, may significantly lower their aesthetic appeal [4,5].

To date, nine species are so far included in the genus *Anisakis* [4]. They are heteroxenous parasites of marine organisms, in which crustaceans (krill) act as first intermediate hosts, fishes and squids as intermediate and/or paratenic hosts and, finally, cetaceans serve as definitive ones [4]. The larval stages of *Anisakis* spp. commonly infect the viscera and musculature of many teleost species [4,6]. These parasites represent a public health concern causing anisakiasis, an emerging fishborne parasitic zoonosis, originating from the consumption of raw or insufficiently thermally processed seafood, carrying alive larvae [4,7,8]. In some South American countries such as Peru, Chile, Ecuador, or Colombia, the higher risk for anisakiasis has been associated to the consumption of traditional raw fish-based dishes, such as ceviche, or insufficiently thermally processed seafood (i.e., salted or marinated) [9,10,11]. Despite those feeding habits, a few human cases have been so far reported in South American countries, mostly from Peru and Chile [12,13,14,15]. However, in those cases, the etiological agent was not identified at the species level. Along the Peruvian coast, the presence of *Anisakis* larvae infecting several fish species with commercial importance (e.g., *S. japonicus*, *T. murphyi*, *S. chilensis*, *C. hippurus*, *Merluccius gayi peruanus, B. japonica*, and *S. violacea*) [16,17] has been reported. Likewise, the adult stage of *Anisakis* spp. has been found in two cetacean species from the same geographic area, *Lagenorhynchus obscurus* and *Phocoena spinipinnis* [18,19]. Some authors [20,21,22] have pointed out that the level of parasitic infection with *Anisakis* spp. larvae observed in *T. murphy*, *S. japonicus*, and other pelagic marine fish from Peruvian and Chilean fishing grounds, can be used as biological tags for the identification of fish stocks along the southeastern coast of the Pacific Ocean [23,24,25].

Unfortunately, those reports of *Anisakis* spp. larvae were so far based on morphologic features of larval stages, lacking certainty in their taxonomic identification, and jeopardizing the knowledge of the biodiversity and distribution of the species of *Anisakis* in the understudied region. Indeed, larval stages of *Anisakis* spp. cannot be recognized at the species level by morphological characters; thereby, the amount of information concerning Peruvian waters is still poor and requires further investigation. It has been underlined that the precise identification of parasites of the genus *Anisakis* is essential for understanding their distribution and epidemiology [4].

The present study aimed to (i) identify *Anisakis* spp. larvae from different economically important fish species collected from two fishing grounds of the Southeastern Pacific Ocean (off the Peru coast) using a genetic-molecular approach; (ii) improve the epidemiological data of *Anisakis* species genetically identified for local fisheries, according to fish species, fishing ground, and site of infection.

## 2. Results

### 2.1. Identification of Anisakis spp.

*Anisakis* spp. larvae (N = 305) collected from the examined fish species were first assigned morphologically (*sensu* Berland, 1961) to the larval morphotype Type I (N = 288) and to the Type II (N = 17). The overall results obtained of the 348 examined fish are reported in Table 1.

According to the sequence analysis at the mtDNA *cox*2 gene locus, 288 *Anisakis* specimens were identified as *A. pegreffii*. Indeed, the specimens matched the 98–100% with the *A. pegreffii* sequences previously deposited in GenBank [26,27] (Table 2).

**Figure 1:**
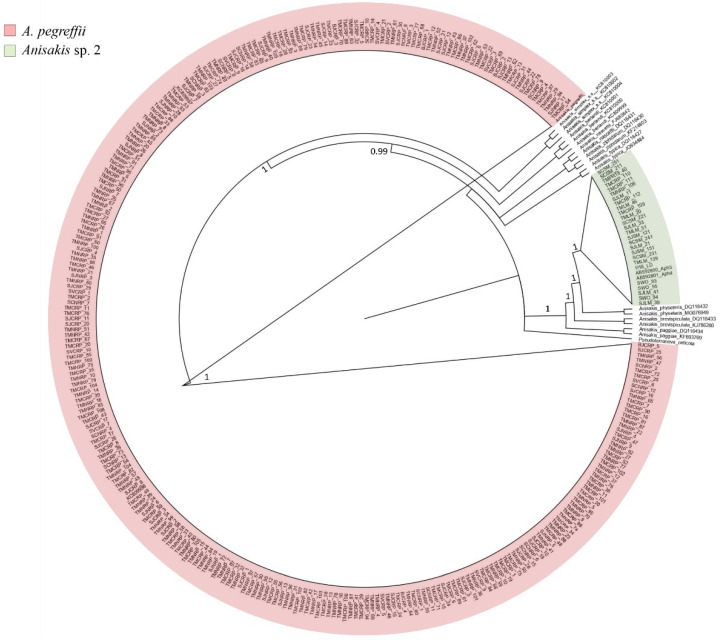
Bayesian inference (BI) circular tree-based on mtDNA *cox*2 gene sequences of *A. pegreffii* and *Anisakis* sp. 2 (larvae obtained from fish species sampled in two fishing ground off the Peruvian coast). The analysis was performed by MrBayes 3.1 [28], using the GTR + G substitution model, as implemented in jModeltest2.1 [29]; the parameter for the selected model was G = 0.131, calculated with Akaike information criterion (AIC) [30]. For the Bayesian analysis, four incrementally heated Markov chains (using default heating values), were run for 1,000,000 generations, sampling the Markov chains at intervals of 100 generations. Numbers at the nodes are posterior probabilities. *Pseudoterranova ceticola* was used as an outgroup.

The phylogenetic tree resulted from the BI analysis (Figure 1) showed that those sequences obtained at the mtDNA *cox*2 gene from those *Anisakis* Type I larvae clustered in a well-supported phylogenetic lineage with a 100% posterior probability value, with the sequences of *A. pegreffii*, previously obtained and deposited in GenBank at that gene.

**Figure 2:**
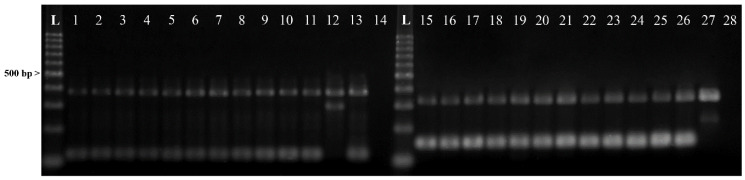
Products (genotypes) of tetraprimer ARMS-PCR obtained at the *nas*10 nDNA locus on larvae of *A. pegreffii* identified in the present study, using the set-1primers, showing: specimen No. 1–10: *A. pegreffii* genotype (bands size: 373-117 bp); 11: positive control of *A. pegreffii*; 12: positive control of *Anisakis simplex* (s.s.) (bands size: 373-296 bp); 13: positive control of *Anisakis berlandi* (bands size: 373-117 bp); 14: negative control. Using the set-2 primers, showing: specimen No. 15–24: *A. pegreffii* genotype (band size: 321-148 bp); 25: positive control of *A. pegreffii*; 26: positive control of *A. simplex* (s.s.) (band size: 321-148 bp); 27: positive control of *A. berlandi* (bands size: 321-216 bp); 28: negative control; L: 100 bp ladder.

In addition, all the specimens identified as *A. pegreffii* (N = 288) by mtDNA *cox*2 sequences analysis, showed a genotype pattern belonging to the *A. pegreffii* species at the tetra-primer ARMS-PCR method, having the diagnostic nucleotide positions at 373 and 117 bp as those given by Palomba et al. [31] for the species *A. pegreffii*. Further, no heterozygote genotypes at those positions were found in the samples of *Anisakis* spp. tested in this study (Figure 2).

**Figure 3:**
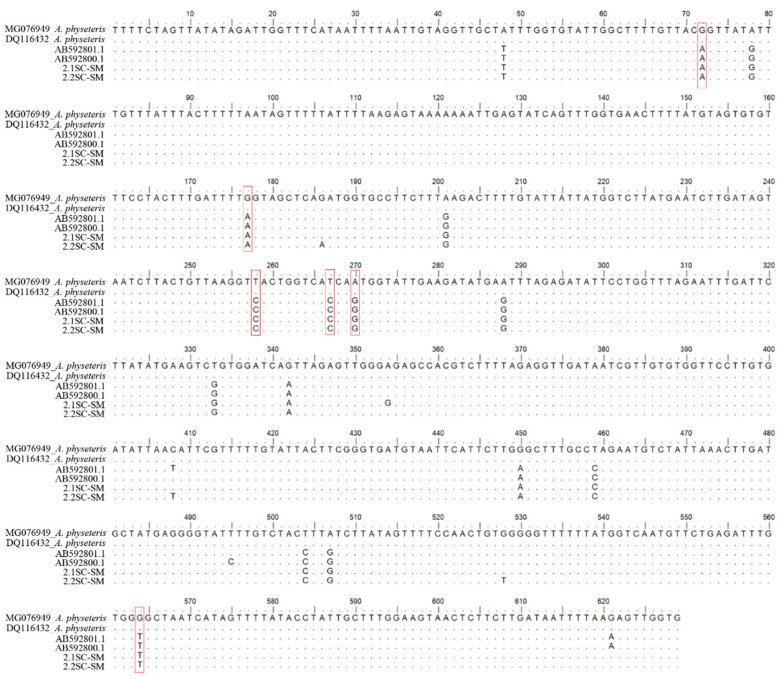
Alignment of mtDNA *cox*2 sequences (629 bp) of *Anisakis* sp. 2 here sequenced with respect to those previously deposited in GenBank by Murata et al. [32] (AB592800; AB592801), and with respect to the consensus sequences of *Anisakis physeteris* (i.e., MG076949; DQ116432) previously obtained at the same gene. The red columns show the fixed substitutions at the nucleotide positions between the two genotypes of *A. physeteris* (s.l.).

The sequences obtained at the mtDNA *cox*2 gene locus from the Type II larvae (N = 17) were quite distinct (96% of genetic similarity) from the reference sequences of *A. physeteris* deposited in GenBank (i.e., DQ116432; MG076949; MG076948; AB592798; KY595213) [26,32]; indeed, they showed six fixed base substitutions at the nucleotide position 73, 177, 258, 267, 270 and 564 (Figure 3) in comparison with those of *A. physeteris*. Instead, those same sequences (N = 17) had a 99.48% of genetic similarity with respect to the mtDNA *cox*2 sequences AB592801 and AB592800 of a genotype of *A. physeteris* (s.l.) previously deposited in GenBank by Murata et al. [32], and indicated by the authors as corresponding to a possible distinct lineage from *A. physeteris* (s.l.) [32]. The phylogenetic inference (BI) here obtained (Figure 1), showed that the sequences from those *Anisakis* Type II larvae are indeed forming a well-supported clade, with a high posterior probability value, including also those two sequences AB592801 and AB592800 (indicated as Aph3 and Aph4 isolates in Murata et al. [32]). In addition, the same sequences of Type II larvae here obtained are clustering in the same clade (Figure 1) with those previously obtained (indicated as SWO93; SWO94 and SWO95) from *Anisakis* Type II larvae detected in the swordfish *Xiphias gladius* from equatorial Atlantic waters [33,34], and indicated as *Anisakis* sp. 2, as likely corresponding to a new genotype of *A. physeteris* (s.l.) (Table 2).

The mtDNA cox2 sequences obtained for the species of *A. pegreffii* are deposited in GenBank under the following Accession numbers: MW074865 and MW074866; while those of *Anisakis* sp. 2 under the following Accession numbers: MW074867 and MW074868.

### 2.2. Distribution of Anisakis spp. by Fishing Ground, Fish Species, and Site of Infection

The overall prevalence of *A. pegreffii* from the two fishing grounds along the Peruvian coast was 29.1% (CI 24.5–34.0) and the mean abundance was 0.83 (±2.22); whereas, *Anisakis* sp. 2 larvae showed a significantly lower prevalence (6.0% (3.6–9.5)) and mean abundance (0.06 (±0.24)) values. *A. pegreffii* larvae were detected both in the viscera and flesh of the examined fish. The two *Anisakis* species co-infected the same individual fish in the viscera of jack mackerel, chub mackerel, and Pacific bonito. No larvae of *Anisakis* sp. 2 were found to infect the fish fillets. Data on the prevalence and mean abundance of *A. pegreffii* and *Anisakis* sp. 2 larvae by the site of infection (viscera cavity and flesh) of the fish species sampled from the two fishing grounds off the Peruvian coast are given in Table 3.

The overall levels of infection with *A. pegreffii* in the *T. murphyi* from the northern coast of Peru exhibited the highest parasitic load, both in prevalence (60.0%) and mean abundance (5.25 ± 5.44), in comparison with the sample from the central coast that showed low parasitic infection rate (P = 39.4%; A= 1.09 ± 1.09). Significant differences in terms of prevalence were observed (*p* = 0.001); however, no significant differences concerning the mean abundance (*p* = 0.45) were found.

**Figure 4:**
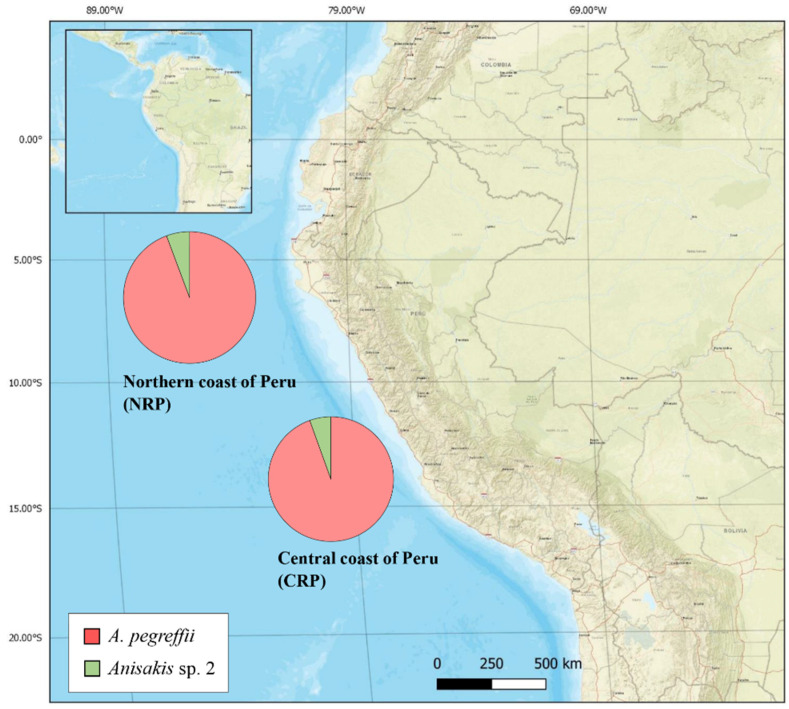
Overall distribution of *A. pegreffii* and *Anisakis* sp. 2 larvae genetically identified in fish species from two fishing grounds (NRP and CRP) of the southeastern Pacific waters (off the Peru coast).

The parasitic load values with *A. pegreffii* in *S. japonicus* caught off the northern (P = 26.1 and A = 0.28) and central (P = 24.6 and A = 0.46) coast of Peru, showed no significant differences between fishing grounds (*p* = 0.40 for prevalence and *p* = 0.35 for abundance). Further, the levels of infection with *Anisakis* sp. 2 from the northern (P = 4.3 and A = 0.04) and central (P = 7.2 and A = 0.07) also showed no significant differences between both areas (*p* = 0.30 for prevalence and *p* = 0.15 for abundance). Regarding the other fish species, in *S. violacea* and *B. japonica,* caught off the central coast of Peru, only *A. pegreffii* larvae were identified. The distribution of *Anisakis* spp. larvae genetically identified from fish species aforementioned along the Peruvian coast are given in Figure 4.

Concerning the site of infection, the parasitic load of *A. pegreffii* larvae was significantly higher in the musculature of the jack mackerel (P = 30, A = 0.50) than that observed in the chub mackerel from the northern coast (P = 4.3, A = 0.04) (respectively *p* = 0.001 for prevalence, and *p* = 0.03 for abundance) (Table 3). Despite the average smaller size of the jack mackerel and chub mackerel, the fish length (TL) did not differ significantly between the two fishing areas (*p* = 0.65 for the northern coast and *p* = 0.57 for the central coast. A significant correlation (r^2^ = 0.18, *p* = 0.0001) between the fish length and the number of larvae resulted in a Spearman correlation analysis, with the total number of larvae increasing with the fish length. In particular, a significant correlation was recorded between fish length and the number of larvae infecting the flesh (r^2^ = 0.19, *p* = 0.0001), and those found in the viscera (r^2^ = 0.11, *p*= 0.0001). Furthermore, a significant correlation was recorded between the value of the parasitic abundance observed in the flesh with those detected in the viscera (r^2^ = 0.36, *p* = 0.0001).

## 3. Discussion

The marine ecosystem off the Peruvian coast represents an important area of exploitable fish biomass of the Southeastern Pacific Ocean region. This area currently yields more than 20 times the tonnage of fishery landings produced by other comparable regional large marine ecosystems that operate under a similar dynamic context, and is characterized by basic primary production [35,36]. The large concentration of pelagic fishes influences the patterns of abundance, spatial distribution, and trophic ecology of a great diversity of top-level predators, including marine mammals and sea birds [37,38], which are definitive hosts of anisakid nematodes [6]. For this reason, the knowledge about the composition and diversity of anisakid parasites distribution in different fish species from this geographic area enhances the possibility of the use of these parasites as indicators of fish host biology, feeding behavior as well as fish stocks composition and migration, and host phylogenetics and systematics [39]. Besides, the presence of nematodes belonging to the genus *Anisakis,* with their zoonotic potential, puts at risk of infection the coastal human populations of Peru, due to their high rate of consumption of raw or undercooked seafood products [4,8,11].

The zoonotic species *A. pegreffii,* identified for the first time in these areas by molecular markers, was detected in commercially important fish species caught in two fishing grounds along the Southeastern Pacific Ocean region off the Peruvian coast. This parasite proved to be the predominant anisakid species, and highly capable of infecting the fish fillets. The finding of *A. pegreffii* in *T. murphyi*, *S. japonicus*, *S. chiliensis*, *S. violacea*, and *B. japonica* represents new host records for this parasite species, and it enlarges its range of distribution to the eastern coast of Pacific Ocean waters from off the Peruvian coast. In addition, in this study, we have further demonstrated the utility of the *nas*10 nDNA nuclear gene as a molecular tool in the identification of the species of the *A. simplex* (s.l.) complex [31].

The data on parasitic burden here observed for the species *A. pegreffii* resulted congruent with previous data for the same parasite collected in other sea basins included in the geographical range of the species. For example, in the Mediterranean Sea, *A. pegreffii* represents the most common anisakid species, and it is found in all pelagic and demersal fish species, often with infection levels comparable to the present findings [40,41,42]. Levsen et al. [5] mentioned that *A. pegreffii* seems to occur occasionally in waters of the northern hemisphere (Northeast Atlantic Ocean). The distribution of *A. pegreffii* is also widespread in the Austral region, between 30° S and 60° S, both in the larval and adult stages [4]. A high prevalence of the parasite species in fish from the East China Sea, the Taiwanese Sea, and the Korean Sea has been also observed [43,44,45,46,47]. Along the coast of Peru, the presence of small cetaceans belonging to the family of Delphinidae is frequently reported. These taxa are listed amongst the main definitive hosts for the species of the *A. simplex* (s.l.) complex in the austral and boreal hemisphere [4,48]. These cetacean species would play their role as definitive hosts of *A. pegreffii* in the Southeastern Pacific Ocean region (off the coast of Peru). Indeed, some authors reported the presence of *Anisakis* sp. infecting the stomach of some delphinids, such as the dusky dolphin, Burmeister’s porpoise, and the long-beaked common dolphin (*Delphinus capensis*) [18,49,50]; however, no identification to species level was performed by those authors. Garcia-Godo et al. [51] also pointed out that the feeding habits of these cetaceans are based mainly on pelagic fish species.

In the present study, the sequences analysis of the mtDNA *cox*2 and their Bayesian inference have shown the possible existence of a further phylogenetic lineage, here indicated as *Anisakis* sp. 2. This genotype, represented by the *Anisakis* Type II larvae collected from the jack mackerel, chub mackerel, and Pacific bonito, seems to be very closely related to the species of *A. physeteris*. This phylogenetic lineage corresponds to the same detected by Murata et al. [32] from Japanese waters, as well as to the same taxon previously discovered by allozyme data in the swordfish from the equatorial area [33,52], and there named as *Anisakis* sp. 2, distinct from *A. physeteris* (s.l.). The specimens indicated *Anisakis* sp. 2 detected in the present study resulted to be indeed a divergent lineage at the mtDNA*cox2* level. *Anisakis* sp. 2 is clustering in the same main clade formed by *A. physeteris*, *A. brevispiculata* and *A. paggiae*. Similar results were gathered by Murata et al. [32]. This finding seems to suggest that *Anisakis* sp. 2 would represent a new gene pool in the *A. physeteris* (s.l.) complex of species. However, the genetic investigation of further nuclear and mitochondrial gene loci is needed to clarify its phylogenetic status and taxonomic position as included in the *A. physeteris* (s.l.) complex of species.

The finding of *A. physeteris* (s.l.) larvae in *T. murphyi*, *S. japonicus*, and *S. chiliensis* from the northern and central coast of Peru also represents new record. The main definitive host of *A. physeteris* species is the sperm whale (*Physeter microcephalus)*, even if adults of the species *A. physeteris* have been also identified in other physeterid, such as *Kogia* spp. [53]. Adults showing the same genotype of *Anisakis* sp. 2 have been also found at the adult stage in an individual of sperm whale from the central Atlantic Ocean Mattiucci et al. (unpublished). Along the Peruvian coast, the presence of the sperm whale has been also reported [48]. Céspedes et al. [54] have detected 50% of prevalence of *Anisakis* Type II larvae in the jumbo squid from the southern coast of Peru, suggesting that it could be an intermediate/paratenic host in the life cycle of the parasite in this geographic area, since it represents a prey item of sperm whale, a main definitive host of the *A. physeteris* (s.l.) [4].

A significant difference was found in the infection level regarding the fishing grounds and the site of infection with both *Anisakis* species. For instance, the overall parasite burden of *A. pegreffii* in jack mackerel reported on the central coast of Peru was significantly lower than on the northern coast of Peru (Table 3). *A. pegreffii* exhibited higher values of infection than *Anisakis* Type II larvae of all fish specimens from the two fishing grounds; the overall higher infection levels are generally similar to those previously reported in European hake (*Merluccius merluccius*) from the Mediterranean Sea [55,56]. Furthermore, *A. pegreffii* larvae occurred in the viscera and flesh of the fish species; whereas, the larvae of *Anisakis* sp. 2 were located in the viscera. The relative proportion of *A. pegreffii* larvae infecting the fish flesh was 5.3% of the total number of larvae collected, which was lower than those detected in the visceral cavity or embedded on the surface of the visceral organs. Similar proportions have been also observed in previous studies carried out on the species *A. pegreffii* [56,57].

Likewise, jack mackerel from the northern coast of Peru showed statistically significant higher prevalence in comparison with those from the central coast. This difference in the prevalence of infection could be explained by the larger body length recorded for the fish obtained off the northern coast compared to the batch originating from the central one. In all the fish samples examined, larger fish tended to show a higher abundance of the parasite. The higher prevalence in larger fish could be explained by parasite accumulation during life [58], considering that *Anisakis* spp. larvae accumulate in the fish host, where they may remain an undetermined time. The correlation between the size of the fish and the parasitic burden is a direct consequence of the time that the fish spent feeding on its prey [4]. The jack mackerel of the northern region recorded higher levels of infection with *A. pegreffii* compared to the fish of the same size range obtained from the central fishing ground. Indeed, Cipriani et al. [56] noted that the level of infection by different species of *Anisakis* varies depending on the fishing ground of the fish host. The infection levels of *Anisakis* species observed in a given geographic area can also be affected by several drivers that shape the population size of the intermediate/paratenic hosts that participate in the life cycle of the parasite [4,59,60]. However, not only biotic factors, but also abiotic parameters, such as sea water temperature and salinity, influence the biogeography and infection dynamics of *Anisakis* species [4].

Since the Peruvian fishery is one of the most important economic activities on the Pacific coast of South America, this study also offers a crucial food safety background for assessing the risk associated with those parasites in seafood products. The fillets, as an edible part of the fish, represent the real risk of the consumer when harboring zoonotic *Anisakis* spp. larvae. This aspect raises in importance especially for the coastal population along the Southeastern Pacific Ocean coast (Colombia, Ecuador, Peru, and Chile) due to their feeding habits of eating dishes based on raw fish. The fish species mainly consumed are the jack mackerel, chub mackerel, and the Pacific bonito. They are all used in preparation of raw-based fish dishes such as “ceviche” and marinated products. For this reason, the exact information on the *Anisakis* larvae location (site of the infection) in the fish host assumes a great importance in a detailed risk assessment for the industry to alert and protect consumers. The presence of *A. pegreffii* on the edible parts of the fish from Peruvian waters not only could represent a zoonosic risk (human anisakiasis) if alive larvae are ingested, but even dead larvae could provoke allergic symptoms in sensitized patients [8]. Indeed, *A. pegreffii* is well known to provoke gastric, intestinal and gastroallergic anisakiasis [4,61]. In addition, massive infections, such as those observed in larger fish, can reduce the market appeal to the consumers. Therefore, an accurate examination of the fish flesh allows a real assessment of the parasitic infection levels to evaluate the real threat of human infection. Finally, the correct genetic/molecular identification of the anisakid nematodes involved in the fish infection represents the basis for any epidemiological survey intended to identify the zoonotic species involved and enhances the possibility to provide an accurate human risk assessment.

## 4. Materials and Methods

### 4.1. Fish Samplings

A total of 348 fish specimens were collected from two fishing grounds along the Peruvian coast, spanning from July 2017 to February 2018. A total of 113 fish specimens (*T. murphyi*, *S. japonicus*, and *S. chiliensis*) were sampled from off the coast of the district of Piura, located on the northern region of Peru (NRP) (04°54′ S, 81°21′ W), whereas 235 fish (*T. murphyi*, *S. japonicus*, *S. violacea*, and *B. japonica*) from off the coast of the district of Lima, located in the central region of Peru (CRP) (12°09′ S, 77°28′ W). The fish were obtained directly from fishing craft; they were immediately frozen and shipped by a refrigerated truck to the Laboratory of Parasitology at the Faculty of Veterinary Medicine & Animal Science of “Universidad Peruana Cayetano Heredia”. The fish were then kept frozen at −20 °C, until their parasitological examination.

### 4.2. Parasitological Analysis

The fish were measured (total body length, TL, 5 mm accuracy) and weighed (total body weight, TW, in g) before inspection for nematode larvae. The fish were gutted, and the viscera of each individual were separated and then carefully observed with a stereomicroscope (Leica DVM6S, Germany). Each fish was manually filleted on both the left- and right-side flesh (butterfly fillets) and placed into clear plastic bags. The samples were then pressed to 1–2 mm thick layers in a hydraulic pressing device and stored overnight at −20 °C for subsequent UV-based detection of larvae, optimizing the quantification of the whole larvae [5,57,62]. Larvae were counted and assigned to the genus level according to diagnostic morphological keys [63], using an optical microscope (Leica DM750, Germany). Finally, the collected larvae were washed in saline solution, fixed in 70% ethanol, and delivered to the Laboratory of Parasitology, Department of Public Health and Infection Diseases of “Sapienza-University in Rome”, where molecular identification was performed.

### 4.3. Genetic Identification of Larval Nematodes

A subsample (N = 305 *Anisakis* spp. Larvae, out of the total 421 collected), corresponding to 72% of the larvae recovered in all the fish specimens from the different fishing grounds, was randomly selected for the identification study by the direct sequence analysis of mitochondrial gene locus mtDNA *cox*2, (629 bp) [27] which allowed us to distinguish all the species so far included in the genus. However, because the Southeastern Pacific Ocean would be a geographic area where the sibling species of the *A. simplex* (s.l.) complex (i.e., *A. simplex* (s.s.), *A. pegreffii*, *A. berlandi*) would occur in sympatry [4], the analysis of a nuclear marker is important for the detection of possible hybrid genotypes between those species. In this regard, the analysis of a nuclear marker, i.e., the *nas*10 nDNA, recently discovered as a diagnostic genetic marker between the three *Anisakis* species of the *A. simplex* (s.l.) complex [31], was also analyzed. In particular, the ARMS-PCR assay of the *nas*10 nDNA [31] was applied to the same specimens previously identified as *A. pegreffii* by mtDNA *cox*2 sequences analysis.

The samples fixed in alcohol were first washed in PBS and distilled water before the DNA extraction. The total DNA of each single larvae was extracted using the *Quick*-gDNA^TM^ Miniprep Kit (ZYMO RESEARCH). The mitochondrial cytochrome C oxidase subunit II (*cox*2) gene was amplified using the primers 211F (5′-TTTTCTAGTTATATAGATTGRTTYAT-3′) and 210R (5′- CACCAACTCTTAAAATTA TC-3′) [26,27]. Polymerase chain reaction (PCR) was carried out according to the previously described procedures [27]. The sequences obtained at the mtDNA *cox*2 for the larval nematodes were compared with those obtained in other previous studies for the same gene and deposited in GenBank: *A. simplex* (s.s.) (DQ116426), *A. pegreffii* (JQ900761), *A. berlandi* (KC809999), *A. typica* (DQ116427), *A. ziphidarum* (DQ116430), *A. nascettii* (FJ685642), *A. physeteris* (DQ116432), *A. brevispiculata* (DQ116433) and *A. paggiae* (DQ116434).

ARMS-PCR assay of the nuclear metallopeptidase *nas*10 nDNA locus was performed in two PCR reactions simultaneously. The PCR reaction was amplified using the set-1 primers: Out-F1 (5′-TATGGCAAATATTATTATCGTA-3′), Out-R1 (5′-TATTTCCGACAGCAAACAA-3′), In-F1 (5′-GCATTGTACACTTCGTATATT-3′ and In-R1 (5′-ATTTCTYCAGCAATCGTAAG-3′) and, for the set-2 primers, we used the following: Out-F2: (5′-GAAAGACAGGTTCATCTCA-3′), Out-R1 (5′-TATTTCCGACAGCAAACAA-3′), In-F2 (5′-AACGGATATGAATGATCCC-3′), In-R2 (5′-HAAATGAAAGTAGAAAGAATTTAC-3′). PCR conditions and procedures were reported in Palomba et al. [31]. The PCR products were separated by electrophoresis using agarose gel (2.5%) and stained with 20-fold dilutions of the stock (10,000×) solution of Diamond™ Nucleic Acid Dye (Promega, Madison, Wisconsin, USA) for the visualization. The distinct banding patterns were detected by the use of ultraviolet transillumination and the sizes of the fragments were determined by comparison with a 100 bp DNA ladder marker (Promega).

A Bayesian inference (BI) tree, based on mtDNA *cox*2 sequences obtained on the *Anisakis* spp. larvae from different host species and fishing grounds were elaborated including sequences of the same species previously studied and deposited in GenBank. The analysis was performed by MrBayes3.1 [28] using the GTR + G substitution model as implemented in jModeltest2.1 [29]. The parameter for the selected model was G = 0.131, calculated with the Akaike information criterion (AIC) [30].

### 4.4. Statistical Analysis of the Parasitic Infection Parameters

Parameters describing parasite infection, i.e., prevalence (P%) and mean abundance (A) of the infection with the detected species of *Anisakis* were calculated using software Quantitative Parasitology 3.0 [64]. Sterne’s exact 95% confidence limits (or adjusted Wald’s for N > 1000) were calculated for prevalence and bootstrap 95% confidence limits (2000 bootstrap replications) for mean abundance, following Bush et al. [65], Rozsa et al. [66] and Reiczigel [67]. Comparison between the levels of infection of *Anisakis* spp. larvae were calculated by Quantitative Parasitology 3.0 [64] using Fisher’s exact test or exact unconditional test (prevalence—depending on sample size) [68] and bootstrap two-sample *t*-test (mean abundance). Differences were considered significant when *p* < 0.05. Differences in fish body length (TL) between the two fishing areas were analyzed with *t*-tests or Kruskal–Wallis tests, depending on data distribution after testing for normality, separately for the jack mackerel and chub mackerel. Spearman rank tests were run to analyze the relationships between the fish host body size (TL) and the overall abundance with *Anisakis* spp. larvae in the viscera and the flesh separately.

## Figures and Tables

**Table 1:** Number of fish examined (N) from the northern coast (NRP) and central coast (CRP) of Peru, recorded with their total body length, weight, and the total number of *Anisakis* spp. larvae collected (N_coll_), and genetically identified (N_id_).

	N	Mean total body length ± SD (Min-Max) (mm)	Mean Weight ± SD (Min-Max) (g)	N_coll_	N_id_
NRP (4°54’ S, 81°21’ W)					
*Trachurus murphyi*	21	337.71 ± 22.85 (282–375)	309.29 ± 30.91 (267–350)	117	106
*Scomber japonicus*	47	275.27 ± 22.9 (236–345)	253.3 ± 38.56 (192–350)	15	15
*Sarda chiliensis*	45	419.33 ± 20.52 (376–473)	1240.3 ± 226.9 (310–1700)	20	20
			Total	152	141
CRP (12°09′ S, 77°28′ W)					
*Trachurus murphyi*	100	257.82 ± 33.6 (243–383)	298.17 ± 35.72 (220–460)	212	112
*Scomber japonicus*	70	294.07 ± 30.88 (236–358)	281.67 ± 52.04 (192–460)	42	37
*Seriolella violacea*	35	282.67 ± 31.24 (241–379)	275.23 ± 31.7 (189–346)	10	10
*Brama japonica*	30	329.25 ± 29.34 (283–381)	310.62 ± 21.89 (285–382)	5	5
			Total	269	164

**Table 2:** Specimens of *A. pegreffii* and *Anisakis* sp. 2 genetically identified in fish species from the Southeastern Pacific waters (off the coast of Peru). NRP: Northern region of Peru. CRP: Central Region of Peru.

	*A. pegreffii*		*Anisakis* sp. 2
	mtDNA *cox*2	*nas*10 nDNA	mtDNA *cox*2
*Trachurus murphyii*			
NRP	105	105	1
CRP	108	108	4
*Scomber japonicus*			
NRP	13	13	2
CRP	32	32	5
*Sarda chiliensis*			
NRP	15	15	5
*Seriolella violacea*			
CRP	10	10	-
*Brama japonica*			
CRP	5	5	-
Total	288	288	17

**Table 3:** Parasitic infection levels with *Anisakis* spp. larvae, genetically identified in fish species from two fishing grounds along the Pacific coast of Peru. Abbreviations: P—prevalence (± 95% CI) and A—mean abundance (± SD). NRP = Northern coast of Peru; CRP = Central coast of Peru.

Fishing Area/Fish Species	*Anisakis pegreffii*						*Anisakis* sp. 2	
	Overall		Viscera		Musculature		Overall/Viscera	
	P (%)	A	P (%)	A	P (%)	A	P (%)	A
NRP (4°54’ S, 81°21’ W)								
*Trachurus murphyi*	60.0 (0.14–0.41)	5.25 ± 5.44	60.0 (0.36–0.81)	4.75 ± 4.91	30.0 (0.12–0.54)	0.50 ± 0.89	5.0 (0.01–0.25)	0.05 ± 0.22
*Scomber japonicus*	26.1 (0.36–0.81)	0.28 ± 0.50	21.7 (0.11–0.36)	0.24 ± 0.48	4.3 (0.01–0.15)	0.04 ± 0.21	4.3 (0.01–0.15)	0.04 ± 0.21
*Sarda chiliensis*	22.7 (0.12–0.38)	0.32 ± 0.67	22.7 (0.12–0.38)	0.32 ± 0.67	-	-	11.4 (0.04–0.25)	0.11 ± 0.32
CRP (12°09’ S, 77°28’ W)								
*Trachurus murphyi*	39.4 (0.30–0.50)	1.09 ± 2.39	38.4 (0.29–0.49)	0.94 ± 2.13	14.1 (0.02–0.14)	0.15 ± 0.39	4.0 (0.01–0.10)	0.04 ± 0.20
*Scomber japonicus*	24.6 (0.15–0.37)	0.46 ± 0.98	24.6 (0.15–0.37)	0.39 ± 0.77	5.8 (0.02–0.14)	0.07 ± 0.31	7.2 (0.02–0.16)	0.07 ± 0.26
*Seriolella violacea*	17.6 (0.07–0.35)	0.32 ± 0.81	17.6 (0.07–0.35)	0.32 ± 0.81	-	-	-	-
*Brama japonica*	17.2 (0.06–0.36)	0.17 ± 0.38	17.2 (0.06–0.36)	0.17 ± 0.38	-	-	-	-
